# Survival of dogs with glomerular disease in Europe: insights from the European Veterinary Renal Pathology Service

**DOI:** 10.1093/jvimsj/aalag161

**Published:** 2026-08-06

**Authors:** Eric Zini, Francesco Rossi, Silvia L Benali, Alice Nicolo, Donatella Gelli, Barbara Contiero, Francesco Dondi, Luca Aresu

**Affiliations:** Clinic for Small Animal Internal Medicine, Vetsuisse Faculty, University of Zurich, 8057 Zurich, Switzerland; Department of Animal Medicine, Production and Health, University of Padova, 35020 Legnaro, Italy; AniCura Istituto Veterinario di Novara, 28060 Granozzo con Monticello, Italy; Clinique vétérinaire AniCura Zebrasoma, 67100 Strasbourg, France; MYLAV, Laboratorio La Vallonea, 20017 Passirana di Rho, Italy; Clinique vétérinaire AniCura Zebrasoma, 67100 Strasbourg, France; Department of Animal Medicine, Production and Health, University of Padova, 35020 Legnaro, Italy; Department of Animal Medicine, Production and Health, University of Padova, 35020 Legnaro, Italy; Department of Veterinary Medical Sciences, Alma Mater Studiorum-University of Bologna, 40064 Ozzano dell'Emilia, Italy; MYLAV, Laboratorio La Vallonea, 20017 Passirana di Rho, Italy; Department of Veterinary Science, University of Turin, 10095 Grugliasco, Italy

**Keywords:** chronic kidney disease, immunosuppressive treatment, outcome, proteinuria

## Abstract

**Background:**

Although glomerular disorders contribute to chronic kidney disease in dogs, few studies have evaluated the usefulness of renal biopsy for predicting outcome.

**Hypothesis/Objectives:**

Investigate morphologic diagnoses, clinicopathological findings, comorbidities, treatment, and their associations with survival in dogs after renal biopsy.

**Animals:**

One hundred ten client-owned dogs with glomerular disease.

**Methods:**

Retrospective cohort study of medical records from the European Veterinary Renal Pathology Service (2018-2023). Data were obtained before referral for renal biopsy and during follow-up. Survival analysis was performed using the Kaplan–Meier method, followed by log-rank tests. Relative risk was calculated using contingency tables.

**Results:**

Dogs were classified into 3 groups: immune complex glomerulonephritis (ICGN) (61 dogs, 56%), non-ICGN (39 dogs, 35%), and juvenile nephropathy (JN) (10 dogs, 9%). Survival was longer in ICGN dogs than non-ICGN (mean ± SEM: 951 ± 78 days vs. 613 ± 92 days; *P* = .02), with no difference versus JN (1004 ± 169). No survival differences were observed among the morphological subtypes of ICGN. Dogs with amyloidosis had shorter survival than all other morphologic non-ICGN subtypes (384 ± 95 vs. 650 ± 56 days, *P* = .02). Risk of a creatinine increase of ≥50% during follow-up was greater in the JN group (RR = 2.78; 95% CI, 1.60-4.84; *P* = .03). Reduction in proteinuria ≥50% during follow-up did not differ between groups. Immunosuppressive treatment was more frequently administered to ICGN dogs (*P* < .001).

**Conclusions and clinical importance:**

Immune complex glomerulonephritis dogs had better outcomes and slower disease progression than non-ICGN and JN. Proteinuria decreased by approximately 50% within each group over time; however, the retrospective design precludes conclusions regarding the effect of antiproteinuric treatment.

## Introduction

Glomerular disease commonly causes chronic kidney disease in dogs.^1^ However, few studies have evaluated its clinical progression, laboratory test results, treatment response, and prognosis after renal biopsy. A review of 137 dogs with protein-losing glomerular disease reported a poor prognosis for dogs with glomerulonephritis and amyloidosis, with a median survival time of 28 days, although some dogs survived longer than 3 years.^2^ In dogs with glomerular disease, nephrotic syndrome is associated with lower survival in non-azotemic cases. Azotemia, however, is linked to a poorer prognosis regardless of the presence of nephrotic syndrome.^3^ Among glomerular diseases, clinicopathologic features and prognosis have been assessed in dogs with focal segmental glomerulosclerosis (FSGS). The median survival time after biopsy was 258 days (range: 26-1003 days). Shorter survival was observed in dogs with serum creatinine ≥2.1 mg/dL and albumin < 2 g/dL.^4^

More recently, a retrospective study investigated survival in dogs with kidney disease, focusing on diagnoses based on renal biopsy and baseline clinicopathologic variables.^5^ The survival of each morphological subtype was compared to that of FSGS patients. Dogs with glomerular amyloidosis had shorter life expectancies, while those with podocytopathy, membranous glomerulonephritis (MGN), mixed MGN, membranoproliferative glomerulonephritis (MPGN), or mixed MPGN had better outcomes. Regardless of the morphologic subtype, higher age, serum creatinine, and urine protein-to-creatinine ratio (UPC) ratio, along with lower serum albumin at biopsy, were associated with a greater risk of death. However, the causes of death were not reported in that study. The roles of treatment and concurrent diseases were not evaluated.^5^

The aim of this retrospective study was to investigate renal-related survival in dogs undergoing kidney biopsy for suspected glomerular disease in Europe, based on comprehensive morphologic assessment. Data included baseline and follow-up clinicopathologic findings, comorbidities, and treatments.

## Materials and methods

### Clinical data

The European Veterinary Renal Pathology Service (EVRPS) database was searched for canine renal biopsy samples submitted between January 2018 and December 2023. Medical records were retrospectively reviewed. Dogs were eligible if a diagnosis of glomerular disease was established based on light microscopy (LM), immunofluorescence (IF), and transmission electron microscopy (TEM) evaluation of renal biopsy specimens. Baseline clinicopathological and treatment data were obtained from biopsy submission forms completed by referring veterinarians. Follow-up information was collected after renal biopsy. Detailed inclusion criteria, data collection procedures, follow-up methodology, and variable definitions are provided in Appendix.

### Processing and evaluation of renal biopsies

All renal biopsy specimens underwent LM, IF, and TEM examination. Further details regarding the biopsy analysis process are provided in Appendix. Renal lesions were evaluated and classified according to the WSAVA Renal Standardization Study Group criteria.^6^ Based on these findings, the diagnoses were categorized into 3 groups.

The first group, immune complex glomerulonephritis (ICGN), was assigned when TEM confirmed glomerular immune complex deposits. The morphological subtypes within this group included MGN, MPGN, mesangioproliferative glomerulonephritis (MeGN), and sclerosing ICGN. The second group, nonimmune complex-mediated glomerulonephritis (non-ICGN), is defined by the absence of glomerular immune complex deposits on the TEM. This latter group included conditions such as minimal change disease (MCD), amyloidosis, FSGS, and diffuse and global glomerular sclerosis. The third group consisted of juvenile nephropathy (JN) cases.^7^

### Statistical analysis

Clinicopathological variables were compared among the ICGN, non-ICGN, and JN groups. Data distribution was assessed before statistical testing, and appropriate parametric or nonparametric methods were applied. Categorical variables were compared among groups using contingency table analyses with adjustment for multiple comparisons.

Survival time was calculated from the date of renal biopsy to death or last available follow-up. Only renal-related deaths were considered events; dogs alive at study completion, lost to follow-up, or dying from nonrenal causes were right-censored. Survival analyses were performed using Kaplan–Meier methods with post hoc comparisons among groups. Because median survival time was frequently not reached, data are reported as the means ± standard errors of the means (SEMs) using restricted mean survival time estimates.

Changes in serum creatinine concentration and UPC during follow-up were also evaluated. Relative risks and corresponding 95% CIs were calculated where appropriate. Statistical significance was set at *P* < .05. Additional details regarding outcome definitions and statistical procedures are provided in Appendix.

## Results

### Study sample characteristics

Over the 6-year study period, 627 canine renal biopsy samples were submitted to the EVRPS. Of these, 605 (96.5%) had a diagnosis of glomerular disease. Baseline and follow-up clinicopathological and treatment data were available for 110 dogs (18.2%), which were included in the study. Renal biopsy samples were received from 14 different European countries. These included Italy (*n* = 32, 29%), the United Kingdom (*n* = 12, 11%), France (*n* = 11, 10%), Germany (*n* = 10, 9%), Sweden (*n* = 10, 9%), the Netherlands (*n* = 7, 6%), Switzerland (*n* = 7, 6%), Belgium (*n* = 5, 5%), Denmark (*n* = 4, 4%), Spain (*n* = 3, 3%), the Czech Republic (*n* = 2, 2%), Portugal (*n* = 2, 2%), Norway (*n* = 1, 1%), and Hungary (*n* = 1, 1%). The remaining renal biopsies (*n* = 3, 2%) were obtained from Israel.

Among the 110 dogs included, 61 (56%) were diagnosed with ICGN, 39 (35%) with non-ICGN, and 10 (9%) with JN. Table 1 reports the different morphological subtypes of ICGN and non-ICGN. Among the 61 dogs with ICGN, 44 (72%) were purebred, 13 (21%) were crossbred, and the breed was unknown for 4 (7%) dogs. A total of 28 different breeds were represented. The most common breeds were Yorkshire Terrier (*n* = 5, 8%), Shetland Sheepdog (*n* = 5, 8%), Golden Retriever (*n* = 4, 7%), and Labrador Retriever (*n* = 3, 5%). The median age was 6 years (range: 1-13 years). Among the 61 dogs, 23 (38%) were intact males, 13 (21%) were intact females, 11 (18%) were neutered males, and 9 (15%) were spayed females. The sex of 5 (8%) dogs was unknown.

**Table 1:** Morphological subtypes of 61 dogs with ICGN and 39 dogs with non-ICGN.

	No. of dogs (%)
**ICGN**	
** MGN**	35 (58)
** MPGN**	21 (34)
** Sclerosing ICGN**	3 (5)
** MeGN**	2 (3)
**Non-ICGN**	
** Amyloidosis**	16 (41)
** FSGS**	9 (23)
** Minimal change disease**	4 (10)
** Diffuse and global glomerular sclerosis**	3 (8)
** Miscellaneous^a^**	7 (18)

^a^Miscellaneous includes podocytopathy (2), cystic glomerulopathy (2), glomerular lipidosis (2), and hypertensive nephroangiosclerosis with moderate arteriosclerosis (1).

Abbreviations: FSGS = focal segmental glomerulosclerosis; MeGN = mesangioproliferative glomerulonephritis; MGN = membranous glomerulonephropathy; MPGN = membranoproliferative glomerulonephritis.

Of the 39 dogs with non-ICGN, 34 (87%) were purebred, 4 (10%) were crossbred, and the breed of one dog (3%) was unknown. A total of 24 different breeds were represented in the non-ICGN group. The English Setter (*n* = 4, 10%) and Italian Pointer (*n* = 3, 8%) were the most common. The median age was 8 years (range: 6 months-12 years). Among the 39 dogs, 17 (44%) were intact males, 10 (26%) were intact females, 6 (15%) were spayed females, and 5 (13%) were neutered males. The sex of 1 dog (2%) was unknown.

All dogs diagnosed with JN were purebred. Eight different breeds were represented, with the most common being Boxer (*n* = 3, 30%). The median age was 2 years (range: 5 months to 4 years). Among the 10 dogs, 5 (50%) were intact males, and the remaining 5 (50%) were intact females.

Dogs with JN were significantly more likely to be purebred than dogs with ICGN (*P* = .04). They were also younger than dogs with ICGN and non-ICGN (*P* < .001). The study sample characteristics did not significantly differ between dogs with ICGN and non-ICGN.

### Pre-biopsy clinicopathologic data and treatment

Table 2 summarizes the laboratory results. No statistically significant differences were found between dogs with ICGN and non-ICGN for any variable. Compared with those in the other 2 disease groups, the dogs in the JN group had significantly higher serum albumin levels and lower UPC (*P* < .001). The remaining results did not differ.

**Table 2:** Selected clinicopathologic variables evaluated before renal biopsy in dogs with ICGN, non-ICGN and juvenile nephropathy.

Variable	ICGN	Non-ICGN	JN	*P* value
**Creatinine (mg/dL)**	1.3 (0.4-5) [*n* = 55/61]	1 (0.5-7.7) [*n* = 34/39]	1.9 (0.9-7) [*n* = 10/10]	.057
**Urea (mg/dL)**	45 (7-202) [*n* = 34/61]	31.5 (5-105) [*n* = 25/39]	44.2 (21-77) [*n* = 8/10]	.100
**Phosphate (mg/dL)**	5 (3.1-18.6) [n = 34/61]	5 (1.4-16.3) [*n* = 22/39]	5.6 (4.1-17.7) [*n* = 7/10]	.199
**SDMA (μg/dL)**	14.5 (6-40) [*n* = 12/61]	13.5 (6-25) [*n* = 8/39]	18 (16-39) [*n* = 3/10]	.187
**Albumin (g/dL)**	1.9 (0.7-3.7)^b^ [*n* = 55/61]	2 (0.6-3.6)^b^ [*n* = 36/39]	3 (1.9-5.4)^a^ [*n* = 9/10]	.001
**Total protein (g/dL)**	5.3 (2.6-7.9) [*n* = 30/61]	5.1 (3.2-6.7) [*n* = 23/39]	5.5 (4.1-7) [*n* = 7/10]	.413
**UPC**	7.5 (0.9-42.5)^a^ [*n* = 56/61]	6.4 (0.6-30.5)^a^ [*n* = 37/39]	1.6 (0.1-20)^b^ [*n* = 9/10]	.001

Data were reported as median and range in brackets (min-max). N indicates the number of dogs for which the data was available. Abbreviations: ICGN = immune-complex glomerulonephritis; JN = juvenile nephropathy; UPC = urine protein-to-creatinine ratio. Different superscript letters along rows mean significant different values.

Twenty-four of 47 dogs (51%) with ICGN, 16 of 31 dogs (52%) with non-ICGN, and 4 of 8 dogs (50%) with JN were hypertensive. The proportion of hypertensive dogs was uniformly distributed among dogs with ICGN, non-ICGN, and JN (*P* = .99). Comorbidities were identified in 25 of 61 dogs (41%) with ICGN, 18 of 39 dogs (46%) with non-ICGN, and 1 of 10 dogs (10%) with JN (Supplementary Material). The proportion of comorbidities did not significantly differ (*P* = .11), while considering disease category neoplastic diseases were significantly more common (*P* = .004) in dogs with non-ICGN (9 of 18; 50%) compared to dogs with ICGN (2 of 25; 8%) and JN (no dogs). Infectious disease testing was performed in 15/61 (25%) dogs with ICGN, 13/39 (33%) with non-ICGN, and 2/10 (20%) with JN. Tests included serology or PCR for *Anaplasma* spp., *Angiostrongylus vasorum*, *Babesia* spp., *Borrellia burgdorferi*, *Dirofilaria immitis*, *Ehrlichia* spp., *Leishmania* spp., and *Leptospira* spp. Among ICGN dogs, 6/15 (40%) tested positive: 3 with MPGN (2 for *B. burgdorferi*, 1 for *Ehrlichia canis*), 2 with MGN (1 each for *B. burgdorferi* and *L. infantum*), and 1 with sclerosing ICGN (*B. burgdorferi*). In the non-ICGN group, 1/13 (8%) dog with FSGS was positive for *B. burgdorferi*. None of the 2 JN dogs tested were positive. Infectious disease prevalence did not differ significantly among groups (*P* = .09).

Pre-biopsy treatments were given to 26/61 (43%) dogs with ICGN, 9/39 (23%) with non-ICGN, and 1/10 (10%) with JN. Dogs with ICGN or non-ICGN were more likely to receive pre-biopsy treatment than those with JN (*P* = .03), with no significant difference in treatment type. Among dogs receiving antiproteinuric therapy, benazepril was used in 21/26 (81%) ICGN, 6/9 (67%) non-ICGN, and 1/1 (100%) JN dogs (*P* = .58), and telmisartan in 3/26 (12%) ICGN and 4/9 (44%) non-ICGN dogs (*P* = .15). Clopidogrel was administered to 8/26 (31%) ICGN and 2/9 (22%) non-ICGN dogs (*P* = .94). Renal diets were fed to 5/26 (19%) ICGN and 2/9 (22%) non-ICGN dogs (*P* = .99). Additional therapies in ICGN dogs included prednisolone (4/26, 15%), enalapril (3/26, 12%), and spironolactone (2/26, 8%).

### Follow-up treatment

Antiproteinuric treatment was administered to 54 of 58 dogs (93%) with ICGN, 32 of 34 dogs (94%) with non-ICGN, and 7 of 8 dogs (88%) with JN. For dogs with ICGN, an ACEi was prescribed to 36 (67%) dogs (*n* = 25 benazepril; *n* = 11 enalapril). An angiotensin II receptor blocker (ARB; telmisartan) was given to 13 (24%) dogs. A combined therapy of both ACEi and ARB was administered to 5 (9%) dogs. In dogs with non-ICGN, an ACEi was administered to 19 (59%) dogs (*n* = 12 benazepril; *n* = 7 enalapril). ARB was given to 6 (19%) dogs. Combined therapy with ACEi and ARB was administered to 7 (22%) dogs. Among dogs with JN, 4 (57%) were treated with ACEi (all benazepril), and 2 (29%) were treated with ARB. The proportion and type of antiproteinuric treatment did not significantly differ among dogs with ICGN, non-ICGN, and JN.

Based on available medical records, immunosuppressive treatment was given to 43 of 57 dogs (75%) with ICGN and 5 of 32 dogs (16%) with non-ICGN. No dogs with JN received immunosuppressive treatment. Among dogs with ICGN, mycophenolate mofetil was prescribed to 34 (79%) dogs, administered either alone (*n* = 29) or in combination with other immunosuppressive drugs (*n* = 5). Nine (21%) dogs were treated with different immunosuppressive drugs. These included prednisolone (*n* = 3), chlorambucil (*n* = 2), prednisolone combined with cyclosporine (*n* = 2), cyclophosphamide (*n* = 1), and prednisolone combined with cyclosporine and azathioprine (*n* = 1). Two of the 5 dogs (40%) with non-ICGN were treated with cyclosporine. The remaining dogs (20% each) received prednisolone only, prednisolone and mycophenolate mofetil, prednisolone, cyclosporine, and leflunomide. Dogs with ICGN were more likely to receive immunosuppressive treatment than were those with non-ICGN and JN (*P* < .001). Mean survival time did not significantly differ between dogs treated or not with immunosuppressive drugs (874 ± 117 vs 1071 ± 113 days, respectively; *P* = .22, τ = 1565). When the analysis was restricted to dogs with ICGN, no significant difference in survival was observed between those treated with mycophenolate mofetil (1080 ± 113 days) and those receiving other immunosuppressive protocols (1219 ± 205 days; *P* = .55, τ = 1530).

Antithrombotic treatment was administered to 26 of 53 dogs (49%) with ICGN, 16 of 32 dogs (50%) with non-ICGN, and 2 of 7 dogs (29%) with JN. For ICGN cases, clopidogrel was prescribed to 23 (88%) dogs, and aspirin was prescribed to 2 (8%) dogs. One dog (4%) was treated simultaneously with both clopidogrel and rivaroxaban. Among dogs with non-ICGN, clopidogrel was prescribed to 15 (94%) dogs, and rivaroxaban was prescribed to 1 (6%). Both dogs with JN were prescribed clopidogrel. The proportion and type of antithrombotic treatment did not significantly differ among the groups.

### Follow-up of clinicopathological data and survival analysis

At the time of data collection, death due to renal causes was reported in 14 of 30 dogs (47%) with ICGN, 17 of 28 dogs (61%) with non-ICGN, and 3 of 6 dogs (50%) with JN. The remaining 16 of 30 dogs (53%) with ICGN, 11 of 28 dogs (39%) with non-ICGN, and 3 of 6 dogs (50%) with JN either died from non-renal causes or had an unreported cause of death. Additionally, 31 of 61 dogs (51%) with ICGN, 11 of 39 dogs (28%) with non-ICGN, and 4 of 10 dogs (40%) with JN were alive at the end of the study or lost during follow-up.

Among dogs with ICGN that did not survive, renal-related death occurred in 7 of 18 (39%) with MGN, 6 of 14 (43%) with MPGN, and 1 of 1 (100%) with MeGN. In non-ICGN cases, renal causes accounted for death in 10 of 15 dogs (67%) with amyloidosis, 2 of 7 (29%) with FSGS, 2 of 2 (100%) with MCD, and 2 of 2 (100%) with diffuse and global glomerulosclerosis. Renal causes also accounted for death in 1 of 5 (20%) dogs classified as having miscellaneous non-ICGN. Renal causes were also the primary cause of death in 3 of the 6 dogs (50%) with JN.

At baseline, in dogs with ICGN, only serum creatinine differed between renal non-survivors and survivors, with higher median values in non-survivors (2.0 mg/dL, range 1.1-5.0 vs 1.1 mg/dL, range 0.4-4.1, respectively; *P* = .007). In the non-ICGN group, non-survivors had lower median albumin (1.6 g/dL, range 0.6-2.7 vs 2.6 g/dL, range 1.5-3.6; *P* = .001) and total protein (4.1 g/dL, range 3.2-5.8 vs 5.7 g/dL, range 4.3-6.7; *P* = .002), and higher UPC (9.4, range 2-18 vs 3.8, range 0.6-30.5; *P* = .009).

**Figure 1:**
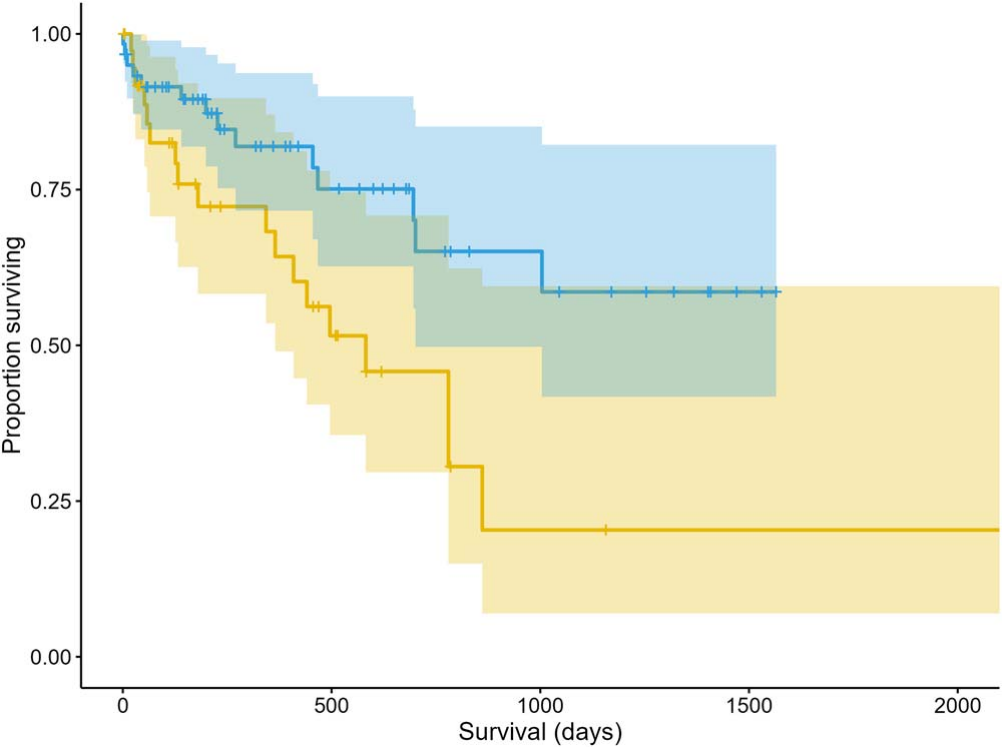
Kaplan–Meier survival curve (and 95% CI) of 61 dogs diagnosed with immune-complex glomerulonephritis (ICGN, blue curve) and 39 dogs with non-ICGN (yellow curve). +: censored data.

Renal-specific survival of dogs with ICGN, non-ICGN, and JN were 951 ± 78 days, 613 ± 92 days, and 1004 ± 169 days, respectively (calculated for τ = 1302). The renal-specific survival of dogs with ICGN was significantly longer compared with non-ICGN (*P* = .02; Figure 1) but did not differ from JN (*P* = .99); no significant difference was observed between non-ICGN and JN (*P* = .13). Among dogs with ICGN, renal-specific survival did not significantly differ between MGN and MPGN (1091 ± 100 vs. 890 ± 165 days; *P* = .30, τ = 1402). The analysis was not performed for sclerosing ICGN and MeGN patients because of the inadequate sample size.

**Figure 2:**
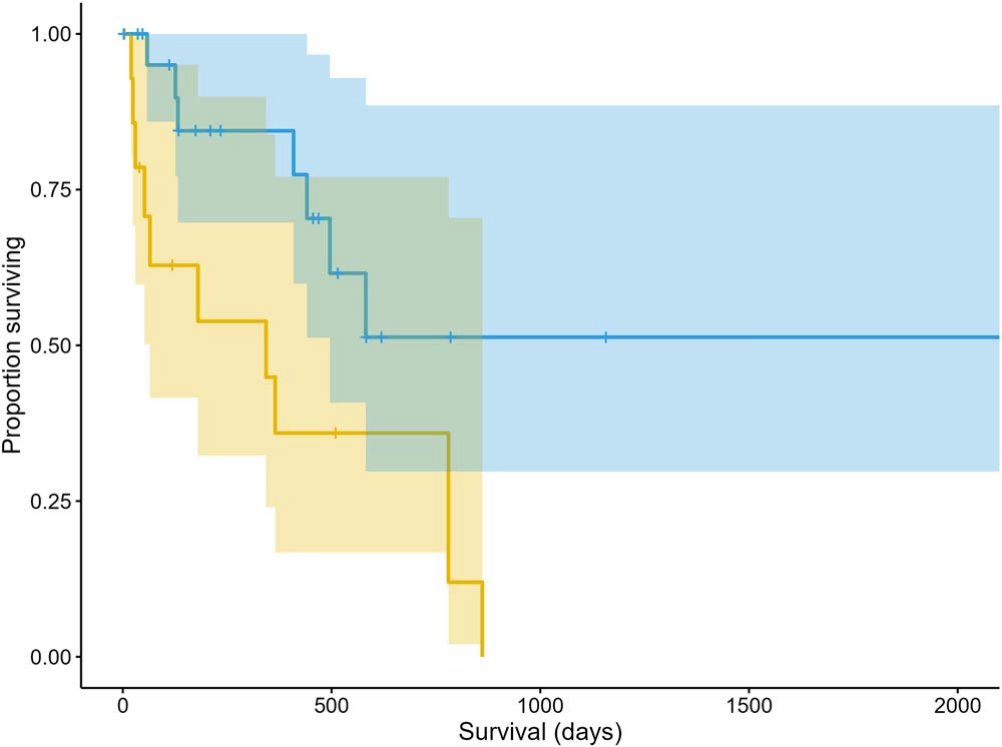
Kaplan–Meier survival curve (and 95% confidence intervals) of 16 dogs diagnosed with amyloidosis (yellow curve) and 23 dogs with other non-immune-complex glomerulonephritis (blue curve). +: censored data.

Among dogs with non-ICGN, renal-specific survival was significantly shorter for those with amyloidosis than for those with any other morphologic subtype of non-ICGN (384 ± 95 vs. 621 ± 70 days; *P* = .04, τ = 861; Figure 2). The second most common morphologic subtype among dogs with non-ICGN was FSGS and the renal-specific survival was significantly longer than in other non-ICGN dogs (904 ± 152 vs. 499 ± 81 days; *P* = .02, τ = 1157).

Overall, 34 of 87 dogs (39%) experienced a serum creatinine increase of ≥50% compared with baseline. The time to reach this endpoint was 966 ± 129 days (τ = 2195). Notably, the RR of a ≥ 50% increase in serum creatinine was significantly greater in dogs with JN than in dogs with ICGN (RR = 2.78; 95% CI, 1.60-4.84; *P* = .03). No significant differences were detected between dogs with ICGN and those with non-ICGN (RR = 0.66; 95% CI, 0.37-1.18; *P* = .25). Similarly, no significant differences were found between dogs with non-ICGN and those with JN (RR = 1.85; 95% CI, 1.09-3.13; *P* = .21). Regarding the time required to reach the time point, no significant differences were found between the 3 groups of subjects (*P* = .16).

Overall, 46 of the 88 dogs (52%) had a UPC decrease of ≥50% compared with the baseline value (required time to reach the end-point: 633 ± 69 days, τ = 1530). These dogs had a significant lower RR of death due to renal causes than those that did not (RR = 0.41; 95% CI, 0.20-0.83; *P* = .01). The proportion of dogs with a UPC reduction of ≥50% did not significantly differ between dogs with ICGN, non-ICGN, and JN [26/52 (50%) vs. 16/29 (55%) vs. 3/7 (43%), respectively; *P* = .83].

Based on data available during follow-up, comorbidities were identified in 34 of 61 dogs (56%) with ICGN, 19 of 39 dogs (49%) with non-ICGN, and 3 of 10 dogs (30%) with JN. Among these dogs, 13 of 34 (38%) dogs with ICGN, 7 of 19 (36%) dogs with non-ICGN, and 2 of 3 (66%) dogs with JN developed a new concurrent disease compared to pre-biopsy period. Overall comorbidities and newly developed concurrent diseases were not significantly different among dogs with ICGN, non-ICGN, and JN (*P* = .30 and *P* = .60, respectively).

The median number of follow-ups was 1 (range: 1-8) for dogs with ICGN, 1 (range: 1-9) for dogs with non-ICGN and 1 (range: 1-6) for dogs with JN; the median time from biopsy to first follow-up was 207 days (range: 25-1170), 150 days (range: 19-782) and 196 days (range: 45-665), respectively.

## Discussion

The results of this study revealed that dogs with ICGN had an approximately 50% longer renal-specific survival than those with non-ICGN. Renal biopsy provides essential information to guide treatment and improve prognostic assessment, supporting individualized management of dogs with glomerular disease. One possible explanation for the improved outcome of ICGN is that, in addition to standard treatment for glomerular diseases, these dogs received immunosuppressive therapy. This therapy targets the formation of immune complexes, which could dissolve and lead to partial recovery from glomerular injury.^8^ In line with this rationale, dogs with ICGN were more likely to receive immunosuppressive treatments. However, as biopsy findings represent injury patterns with variable underlying causes and clinical courses, longer survival in ICGN dogs might have resulted from less severe disease at the time of biopsy compared with non-ICGN dogs.

Few studies have evaluated the value of morphological subtypes of glomerular disease for predicting outcome in dogs.^2^^,^^5^^,^^8^ As in our findings, in the most recent investigation from the United States,^5^ improved outcomes were reported for MGN and MPGN, both of which are ICGN. However, in that study it is not specified whether cases of MGN and MPGN had received immunosuppressive treatment, thus making it unclear if it played a role on life expectancy. The same study also reported improved outcomes for podocytopathy, which is non-ICGN. Of note, in our series podocytopathy was reported in 2 dogs, hampering the analysis and possibly highlighting differences in the distribution of morphological subtypes between United States and Europe.

The relationship between pre-biopsy findings of infection diseases and co-morbidities should be interpreted with caution. Neoplastic comorbidities were more frequent in dogs with non-ICGN, but the small sample size and incomplete clinical data limit the robustness of this finding. Similarly, few dogs were tested for infectious diseases, reducing statistical power and likely masking group differences. Data limitations might have influenced the observed associations. Additionally, geographic variations in the prevalence of infectious and vector-borne diseases between European regions, particularly the Mediterranean and southern Europe, and the United States might affect the observed prevalence, characteristics, and treatment of glomerular diseases.^9–13^

Sex hormones influence renal physiology and might contribute to kidney disease pathogenesis. In humans, estrogen and progesterone support renal perfusion, filtration, and tubular function, whereas testosterone has opposing effects, with estrogen providing predominant protection in females.^14^ Reproductive status has also been considered in dogs. In a recent study, 79% of males were neutered and 89% of females spayed among dogs with follow-up data.^5^ In our cohort, when sex status was known, there was no significant difference in sex distribution between ICGN and non-ICGN dogs; overall, 29% were neutered males and 39% spayed females. Thus, the proportion of sterilized animals differed markedly between studies. Although no sex-related differences in disease type were observed, the lower percentage of neutered dogs in our study sample might have influenced disease manifestation or progression. Prospective studies are needed to evaluate the role of reproductive status and sex hormones in renal function and disease development.

Regarding pre-biopsy treatments, the lower frequency in dogs with JN compared to ICGN or non-ICGN likely reflects milder clinicopathologic abnormalities at presentation, including lower proteinuria and higher serum albumin.

Similar to previous investigations, amyloidosis in our series was associated with a poor outcome. However, compared with other studies, survival was on average twice as long (ie, one year versus less than 6 months).^2^^,^^5^^,^^15^ Notably, the association of amyloidosis with chronic inflammatory and neoplastic disorders^16^ suggests that concurrent comorbidities, along with their response to treatment, negatively impact life expectancy. In our cohort, only one-fifth of dogs with amyloidosis had a comorbidity identified before biopsy, and only one developed a comorbidity during follow-up. This low prevalence might have contributed to the more favorable outcomes observed compared to previous reports. Additionally, no dogs with amyloidosis in our study were Chinese Shar-Pei. This breed has a 3-fold higher serum creatinine concentration at presentation than other breeds with renal amyloidosis, and this is negatively associated with survival.^16^ Differences in breed distribution thus also contributed to a less guarded prognosis.

The second most common morphological subtype in dogs with non-ICGN was FSGS, a well-recognized cause of proteinuria.^4^ Among dogs with non-ICGN, dogs with FSGS had significantly longer renal-specific survival, with a life expectancy of approximately one year. This aligns with previous reports of survival ranging from less than 1 to 1.5 years,^4^^,^^5^ and suggests that FSGS might be associated with a better outcome than some other non-ICGN subtypes.^5^

In line with a past publication, dogs affected by JN were younger and presented lower pre-biopsy urine protein loss than those with ICGN and non-ICGN.^7^ Although dogs with JN had a greater risk of creatinine increasing to above 50% from pre-biopsy concentrations than those with ICGN, their overall survival did not significantly differ from that of dogs with either ICGN or non-ICGN. However, the small number of JN cases limits any definitive conclusions. Studies involving larger cohorts of dogs with JN are thus needed to better define their progression and the effects of antiproteinuric treatment.

Higher baseline creatinine and UPC, and lower albumin, were associated with poorer outcomes, consistent with previous reports.^5^^,^^18^^,^^19^ Nearly half of the dogs in this study achieved a proteinuria reduction greater than 50% from pre-biopsy values, regardless of morphologic diagnosis, and these dogs had a lower risk of death, consistent with previous findings.^5^^,^^18^^,^^19^ Antiproteinuric therapy was administered to over 90% of dogs, and inhibition of the renin-angiotensin-aldosterone system (RAAS) using ARB, either alone^18^ or combined with ACEi,^20^ is a standard care for proteinuric dogs. Optimized RAAS blockade can substantially reduce proteinuria^21^ and is associated with improved survival.^5^^,^^16^^,^^17^^,^^19^ As expected in our study, no significant differences were observed in the proportion or type of therapy among the groups. Indeed, antiproteinuric treatment is currently not indicated for one specific disease. Rather, it is indicated for any dog with proteinuria.^20^

The more frequent use of immunosuppressants in dogs with ICGN suggests reliance on biopsy-guided therapy and reinforces the need for histologic diagnosis before immunomodulation. Most dogs with ICGN are treated with mycophenolate mofetil, either alone or in combination with other immunosuppressive treatments. Although survival did not differ significantly between dogs treated or not with immunosuppressive drugs, nor between ICGN dogs treated with mycophenolate mofetil versus other immunomodulators, the small number of cases per treatment subgroup might have limited detection of significant associations. Additionally, the retrospective design precluded access to monitoring variables, such as B-cell counts or other biomarkers, to confirm the degree of immunosuppression achieved. Retrospective assessment of treatment efficacy is also inherently limited by variability in clinical decisions, dosing, and monitoring by referring veterinarians.

However, mycophenolate mofetil is recommended for the treatment of dogs with ICGN.^8^ Nevertheless, prospective comparative studies are warranted to evaluate different immunosuppressive treatments for each morphologic subtype of ICGN. Fewer than one-fifth of dogs in the non-ICGN group received immunosuppressive therapy, occasionally due to concurrent conditions requiring immunomodulation. The use of immunosuppressive treatments in dogs with glomerular disease lacking histopathologic diagnosis should be cautiously considered, with close and careful patient monitoring.^22^ In human medicine, kidney biopsy is essential to guide immunosuppressive therapy in glomerulonephritis, as indiscriminate use can be ineffective or harmful.^23^

Only approximately half of the dogs with ICGN and non-ICGN received antithrombotic treatment. This limited use might reflect clinicians’ concerns regarding bleeding risk or underrecognition of thrombosis risk. Protein losing nephropathy is associated with the development of thrombosis in dogs. Antithrombotic treatment is recommended irrespective of the serum albumin concentration and degree of proteinuria.^24^ Not unexpectedly, comorbidities were common in dogs with ICGN and non-ICGN. They were less common in dogs with JN, probably because of the younger age of the latter group.

This study has some limitations, primarily due to its retrospective nature. Dogs were included based on the availability of follow-up data. This might have led to the preferential inclusion of cases with more intensive diagnostic workups. Additionally, some data were missing. The study was constrained by its sample size, which might have led to imprecision in some estimates; therefore, the findings should be validated in larger cohorts. It is clear that achieving a larger sample size is challenging, especially given that the analysis already spans an extended period of 6 years (2018-2023). Furthermore, the follow-up duration and consistency of the collected clinicopathological variables differed, occasionally even for the same case. Although SBP was reported, it was not analyzed because only a single measurement per dog was available, and details on measurement technique, number of readings, or antihypertensive treatment were lacking. Causes of death were assigned based on referring veterinarians’ reports, so contributions of comorbidities such as hypertension or hyperadrenocorticism might be underrepresented. Despite incomplete data and potential misclassification, the main survival findings are considered reliable, with this limitation likely evenly distributed across morphologic diagnoses. Finally, the biochemistry profiles were obtained from different laboratories, which might have affected some of the results, and the methodology to measure variables such as the creatinine was not available.

In conclusion, renal biopsy provides important diagnostic and prognostic information in dogs with glomerular disease. Dogs with ICGN had longer renal-specific survival than those with non-ICGN, with amyloidosis showing the poorest outcome, while dogs with JN had survival comparable to ICGN despite faster progression. Proteinuria decreased by approximately 50% from baseline in each group, likely reflecting widespread use of antiproteinuric therapy, and was associated with improved survival, highlighting the value of monitoring proteinuria. Immunosuppressive treatment was appropriately prescribed in dogs with ICGN due to immune-complex deposition.

## Supplementary Material

Supplementary_material_aalag161

## Off-label antimicrobial declaration

Authors declare no off-label use of antimicrobials.

## Institutional animal care and use committee or other approval declaration

Authors declare no institutional animal care and use committee or other approval was needed.

## Appendix Detailed materials and methods

### Clinical data collection

The European Veterinary Renal Pathology Service (EVRPS; https://evrps.mylav.net/) provides detailed pathological evaluations of canine and feline kidney samples using light microscopy (LM), transmission electron microscopy (TEM), and immunofluorescence (IF).

Signalment and clinicopathologic data were obtained from biopsy submission forms completed by referring veterinarians (https://evrps.mylav.net/submission-form/). Baseline variables included serum creatinine, urea, phosphate, symmetric dimethylarginine (SDMA), total protein, albumin, urine specific gravity, urine protein-to-creatinine ratio (UPC), systolic blood pressure (SBP), concurrent diseases, and treatments. Systemic hypertension was defined as SBP ≥160 mmHg.^25^

Follow-up data were collected using a standardized questionnaire sent to referring veterinarians every 6 months. Questionnaires included information regarding survival status, cause of death, comorbidities, treatments, serum creatinine concentration, and UPC measurements obtained during follow-up.

Questions included were the following:

1) Is the patient alive?
2) What are the last creatinine, BUN and phosphate serum levels?
3) What are the last SDMA serum levels?
4) What are the last albumin and total protein serum levels?
5) What is the last urine protein-to-creatinine (UPC) ratio value?
6) When were the last blood work and urinalysis performed?
7) Is the patient hypertensive?
8) May you list all drugs with the dosage (per kg of body weight) the patient is currently receiving?
9) Has the patient any concurrent disease, in addition to the renal disorder?

Dogs were monitored until death or loss to follow-up. Dogs were considered lost to follow-up when no additional information was obtained after the last questionnaire distributed. No predefined follow-up endpoint was established.

Dogs were included if baseline data closest to the time of renal biopsy and at least one post-biopsy renal evaluation within 6 months and subsequent 6-month intervals were available. Survival analyses required either a confirmed date of death or follow-up information. Variable-specific analyses required baseline data and at least one subsequent measurement of the variable of interest. Complete blood count data were optional. Cases with missing data were excluded only from analyses involving the missing variable.

### Renal biopsy processing

All renal biopsy specimens underwent LM, IF, and TEM evaluation.

For LM, tissue sections were cut at 3 μm and stained with hematoxylin and eosin, periodic acid–Schiff, Masson’s trichrome, and periodic acid–methenamine silver stains. Congo red staining of 8–10 μm sections was performed when amyloidosis was suspected.

For TEM, tissues were fixed in chilled 3% buffered glutaraldehyde and processed according to standard protocols.

For IF, antibodies directed against IgG, IgM, IgA, and complement component C3 were used.^26^

### Statistical analysis

The normal distribution of the data was assessed using the Shapiro–Wilk test. Data are reported as the mean ± standard deviation (SD) for normally distributed variables or as the median (min-max) for non-normally distributed variables. Comparisons among ICGN, non-ICGN, and JN groups were performed using one-way ANOVA for normally distributed variables and Kruskal–Wallis tests for nonnormally distributed variables. Categorical data, expressed as percentages, were compared among the three groups using a k-proportion test (based on χ-square test). Post hoc pairwise comparisons were adjusted via Bonferroni correction.

For survival analyses, dogs were categorized as still alive, lost to follow-up, dead from renal-related causes, or dead from nonrenal-related causes based on clinicopathologic data provided by the referring veterinarians. The time in days was recorded from renal biopsy to death or the last available follow-up. Only deaths attributed to renal causes were considered. Dogs still alive at the end of the study, dogs lost to follow-up and dogs that died due to nonrenal-related causes were right-censored. By accounting for right-censoring, the analysis properly reflects the time to renal-related events while appropriately handling incomplete information, thus providing unbiased estimates of the risk over time. Life expectancies for ICGN, non-ICGN, and JN patients, as well as for each morphologic subtype within the three groups, were studied using the Kaplan–Meier survival analysis. Pairwise post hoc log-rank analyses were performed to identify significant differences between groups.

In many cases, the median survival time could not be calculated because the survival curve did not drop to or below the 50% mark by the end of the study period. The data are reported as the means±standard errors of the means (SEMs) using the restricted mean survival time (RMST) approach calculated on the largest observed survival time (τ).^27^ The same Kaplan–Meier approach was also used to calculate the time required to reach the endpoint (creatinine increasing ≥50% and UPC decreasing ≥50%).

Additionally, r × c contingency tables were used to estimate the relative risk (RR) of a ≥ 50% increase in creatinine and a ≥ 50% in UPC from baseline among the three groups. The same analysis compared the proportions of dogs with systemic hypertension, comorbidities, or receiving treatments (antiproteinuric, immunosuppressive, or antithrombotic) across groups.

Cases with missing data were excluded from the relevant analyses. All analyses were performed using SAS version 9.4 (SAS Institute Inc., Cary, NC, USA).

## Abbreviations

ACEi: angiotensin converting enzyme inhibitors
ARB: angiotensin II receptor blocker
EVRPS: European Veterinary Renal Pathology Service
FSGS I: focal and segmental glomerulosclerosis not associated with immune-complex deposits
FSGS II: focal and segmental glomerulosclerosis secondary to immune-complex deposits
ICGN: immune complex glomerulonephritis
IF: immunofluorescence
JN: juvenile nephropathy
LM: light microscopy
MGN: membranous glomerulonephritis
MPGN: membranoproliferative glomerulonephritis
MeGN: mesangioproliferative glomerulonephritis
MCD: minimal change disease
non-ICGN: nonimmune complex-mediated glomerulonephritis
RAAS: renin-angiotensin-aldosterone system
TEM: transmission electron microscopy
SDMA: symmetric dimethylarginine
SBP: systolic blood pressure
UPC: urine protein-to-creatinine ratio
